# 2D PEA_2_SnI_4_ Inkjet-Printed Halide Perovskite LEDs on Rigid and Flexible Substrates

**DOI:** 10.1021/acsenergylett.2c01773

**Published:** 2022-09-30

**Authors:** Giovanni Vescio, Jesus Sanchez-Diaz, Juan Luis Frieiro, Rafael S. Sánchez, Sergi Hernández, Albert Cirera, Iván Mora-Seró, Blas Garrido

**Affiliations:** † MIND-IN2UB, Department of Electronics and Biomedical Engineering, Universitat de Barcelona, Martí i Franquès 1, Barcelona 08028, Spain; ‡ Institute of Advanced Materials (INAM), Universitat Jaume I (UJI), Avenida de Vicent Sos Baynat, s/n, Castelló de la Plana 12071, Spain

## Abstract

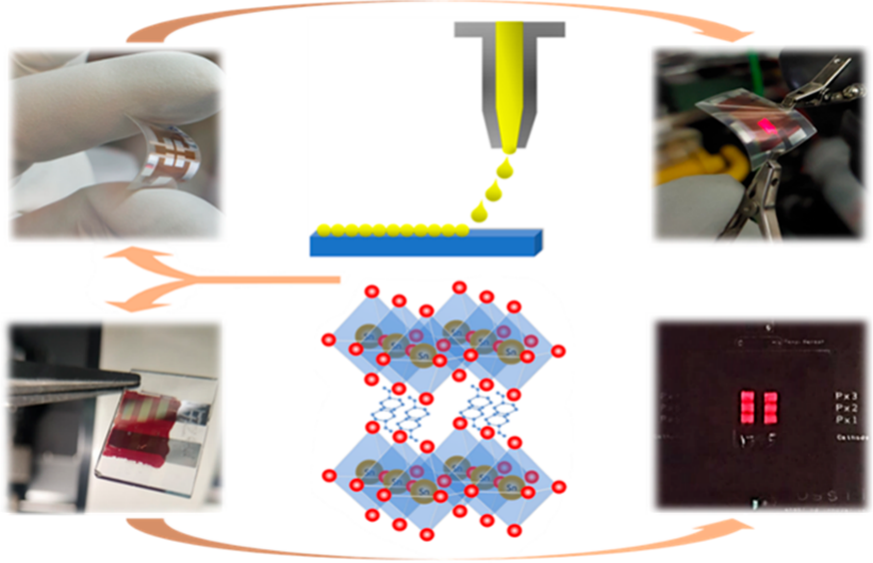

Lead-free PEA_2_SnI_4_-based perovskite LEDs are successfully inkjet-printed on rigid and flexible substrates. Red-emitting devices (λ_max_ = 633 nm) exhibit, under ambient conditions, a maximum external quantum efficiency (EQE_max_) of 1% with a related brightness of 30 cd/m^2^ at 10 mA/cm^2^.

Halide perovskites (HPs) have attracted attention over the past decade due to their excellent optoelectronic properties in the field of photovoltaics (PV) and light-emitting diodes (LEDs).^1,2^ Here, perovskite-based light-emitting diodes (PeLEDs) show external quantum efficiencies (EQE) exceeding 20%.^3,4^ Recently, there has been much work focused on lead-free HPs, mostly in PVs, as the most promising strategies to tackle toxicity issues. However, development of Pb-free PeLEDs has received less attention mostly due to their intrinsic lower stability in comparison with their Pb-containing PeLED counterparts. Consequently, the development of Pb-free PeLEDs fabricated with industrially friendly techniques is a major milestone of the field.

3D HPs present low exciton binding energies and the use of low-dimensional structures, as 2D HPs, is preferred for the fabrication of PeLEDs.^5,6^ As in the case of Pb-free HP PVs, the most promising family for the development of PeLEDs are Sn-HPs. Nevertheless, despite the considerable progress in terms of performance achieved (EQE and luminance),^3,7,8^ Sn^2+^ in its oxidation state is prone to undergo oxidation under ambient conditions, forming its tetravalent state Sn^4+^. This fact causes a *p*-type self-doping process, leaving undesired Sn^2+^ vacancies that act as nonradiative recombination centers, thus quenching the perovskite emission. Several approaches and efforts have been dedicated to overcome Sn^2+^ oxidation.^9^ A few studies validate SnF_2_ as an additive widely used as Sn compensator in solar cells,^10,11^ the introduction of Cl doping,^10^ or the use of a reasonable amount of metal tin.^10^ The use of a NaBH_4_ reducing additive has been shown to be very beneficial in increasing the stability of Sn-based HP PVs.^12^

Beyond materials demands, development of Pb-free PeLEDs will require industrial friendly fabrication methods, since spin-coating, the usual lab approach for film deposition, is not an appropriate technique for upscaling. It does not offer spatial resolution for the deposition of multiple LEDs in large areas, and a major amount of the precursors is wasted.^13,14^ In contrast, inkjet printing is an emerging technology suitable to achieve smooth, uniform, and pinhole free thin films^15^ that can be exploited to produce low-cost, large area, and even foldable devices and arrays.^16^ Inkjet printing is believed to be the most feasible tool for patterning full color QD-LED displays for mass production.^17^

In this Energy Express, we present, to the best of our knowledge, the first report on the fabrication of Pb-Free HP PeLEDs (i) through inkjet printing deposition of the active layer and (ii) on flexible substrates. PeLEDs based on 2D PEA_2_SnI_4_ (PEA, phenylethylammonium) have been fabricated by inkjet-printing technology, on both glass and polyimide (PI) flexible substrates, with red emission (λ_max_ = 630 nm).

The quality of PEA_2_SnI_4_ inkjet-printed layers, without the use of antisolvent, is strongly determined by the incorporation of additives. The top-view scanning electron microscopy (SEM) images reveal a clear improvement with the introduction of different additives; see Figure 1. PEA_2_SnI_4_ films without additives present nonuniform films with abundant pin-holes. Remarkably, the addition of SnF_2_ as an additive reduces the pinhole density; however, the most successful printed layers, without detrimental morphological defects, are those with the inclusion of NaBH_4_, and especially outstanding are those with the combination of SnF_2_ + NaBH_4_. Light absorption determined by photoluminescence excitation (PLE) exhibits a sharp excitonic feature at λ ≈ 621 nm, with a PL emission peak centered at λ_max_ ≈ 633 nm with a narrow fwhm of 30 nm; see Figure 2a.

**Figure 1:**
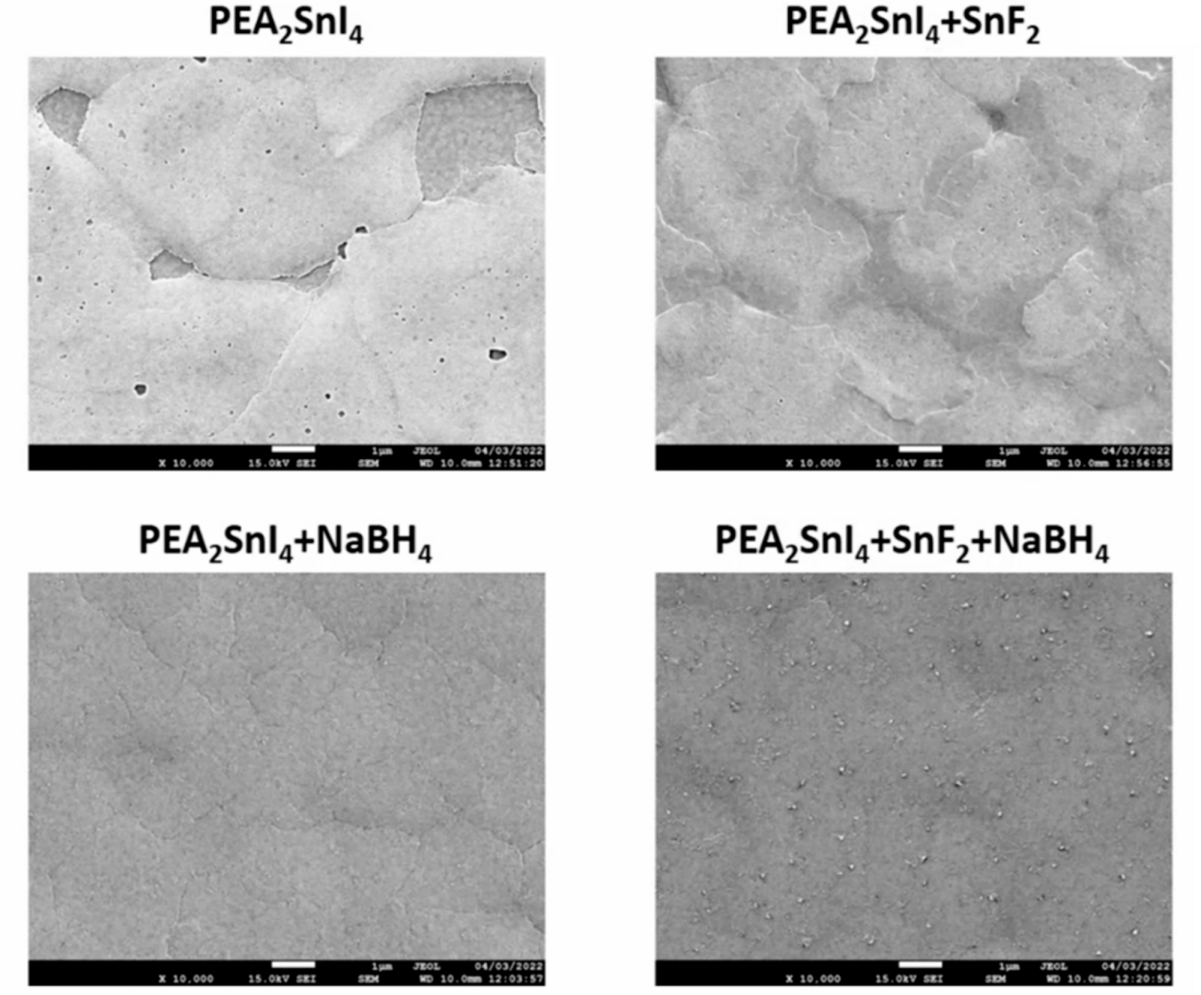
SEM top view images of different inkjet-printed layers by using different additives into the PEA_2_SnI_4_ ink solution.

**Figure 2:**
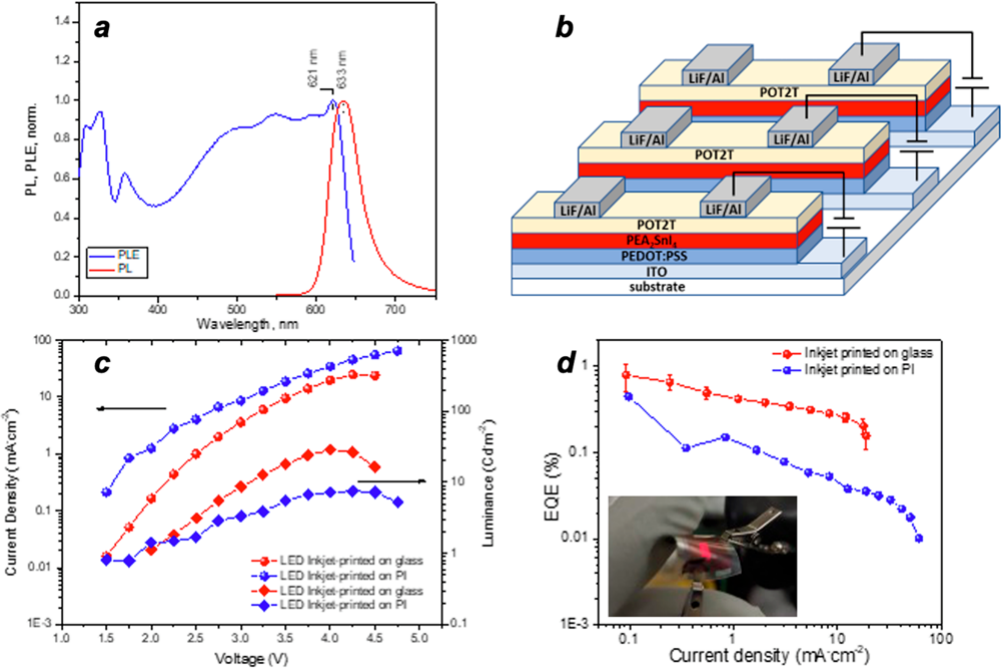
(a) PLE and PL of inkjet-printed PEA_2_SnI_4_. (b) Devices structure. (c) Current density and luminance vs voltage characteristics. (d) EQE vs current density (inset is the image of the red emitting flexible LEDs operating at 5 V).

X-ray diffraction pattern exhibits characteristic (001) reflections of the layered structures; see Figure S1. From Tauc plot analysis, the band gap of the PEA_2_SnI_4_ layer is estimated to be ≈1.89 eV; see Figure S2. We have properly designed the PeLED device with PEDOT:PSS (poly(3,4-ethylenedioxythiophene) polystyrenesulfonate) and PO-T2T (2,4,6-tris[3(diphenylphosphinyl)phenyl]-1,3,5-triazine), as appropriate hole and electron injecting layers; see the energy level diagram, in Figure S3, and the configuration of the fabricated devices, in Figure 2b.

We have manufactured Sn-based PeLEDs through the following process: PEA_2_SnI_4_ thin films (≈ 80 nm) were inkjet-printed onto PEDOT:PSS-coated prepatterned ITO substrates (glass and PI), from a solution containing PEAI and SnI_2_ in a solvent mixture DMF:DMSO (4:1 v/v), with both additive SnF_2_ + NaBH_4_ and followed by vacuum annealing at 100 °C (15 min). The device architecture is completed by evaporating POT2T, LiF, and Al contacts. See Supporting Information for more details. Pictures of the fabricated devices even under working conditions can be found in Figure S4 and in the inset of Figure 2d. Cross-sectional SEM images corroborated the printed Sn-based perovskite layer compactness of our devices; see Figure S5 also for the different layer thickness.

Device characterization experiments were performed under ambient conditions after a proper encapsulation, see Supporting Information. The electroluminescence (EL) spectral characteristics show that the light emitted by the first inkjet-printed PEA_2_SnI_4_ devices, with both SnF_2_ and NaBH_4_ additives, is red with a peak centered at 630 nm with fwhm of 25 nm; see Figure S6. Figure 2c presents the current density and luminance as a function of the operating voltage on both glass and PI substrates. Moving the fabrication of LED devices from rigid to flexible substrate promotes a slight decrease of performances. Similarly, the EQE, plotted as a function of current density, verifies the tendency; see Figure 2d. The maximum EQE is ≈1.0%. This value is very promising and is close to that of the most efficient reported red-emitting lead-free PeLED fabricated by the spin-coating method.^8^ The turn-on voltage is around 1.5 V, and the achieved maximum luminance is 30 cd/m^2^ at 4 V for glass substrate PeLEDs with an efficiency of 0.5 cd/A at a current density of 10 mA/cm^2^. See Video S1 for a demonstration of an array PeLED operation. All the inkjet-printed PEA_2_SnI_4_-based LEDs show a half-lifetime exceeding 3 h.

The presented devices correspond, as far as we know, to the first demonstration of the viability of inkjet printing for the fabrication of lead-free PeLEDs, not only on rigid but also on flexible substrates. The exploitation of this technique ensures a future scalability in area and number of devices for mass production required in industry.

## Supporting Information Available

Experimental procedures, X-ray diffraction, Tauc plot, Energy level diagram, device pictures, SEM cross section of the LED, and caption for a video of an array of 6 LEDs (PDF)
Video of an array of 6 LEDs (MP4)
