# Supplementary material for: 2D PEA2SnI4 Inkjet-Printed Halide Perovskite LEDs on Rigid and Flexible Substrates
Source: ACS Energy Lett. 2022 Sep 30;7(10):3653–5. doi: 10.1021/acsenergylett.2c01773 (PMC9578039; doi:10.1021/acsenergylett.2c01773)
Supplement: Supplementary file 1 — nz2c01773_si_001.pdf [file nz2c01773_si_001.pdf]

Supplementary Materials for

2D PEA_2SnI_4 Inkjet-Printed Halide Perovskite LEDs on Rigid and Flexible Substrates

Giovanni Vescio,[1,†,] Jesus Sanchez-Diaz,[2,†] Juan Luis Frieiro,[1] Rafael S. Sánchez,[2] Sergi Hernández[1] Albert Cirera,[1] Iván Mora-Seró,[2,*] Blas Garrido[1,*]*

[1] MIND-IN2UB, Department of Electronics and Biomedical Engineering, Universitat de Barcelona, Martí i Franquès 1, Barcelona 08028, Spain.

[2] Institute of Advanced Materials (INAM), Universitat Jaume I (UJI), Avenida de Vicent Sos Baynat, s/n, Castelló de la Plana 12071, Spain.

[†] Both authors contribute equally.

Corresponding Authors: gvescio@ub.edu, sero@uji.es, blas.garrido@ub.edu

This Word document file includes:

Figures from S1 to S6

EXPERIMENTAL PROCEDURES

Materials

Tin(II) iodide (SnI_2, 99.99%), tin(II) fluoride (SnF_2, 99%), Sodium borohydride ($NaBH_4$, 96%), N,N-dimethylformamide (DMF, 99.8%), and dimethylsulfoxide (DMSO, 99.8%) were purchased from Sigma-Aldrich. 2,4,6-tris[3-(diphenylphosphinyl)phenyl]-1,3,5-triazine (PO-T2T) was purchased from Lumtec Taiwan. PEDOT:PSS Al 4083 aqueous solution was purchased from Heraeus. Phenylethylammonium iodide (PEAI. 98%) was purchased from Greatcell solar materials. All materials were use as received with no further purifications. Prepatterned ITO glass substrates (6 pixels) were purchased from Ossila. prepatterned ITO flexible polyimide substrates (4 pixels) were purchased from Biotain.

Ink preparation

0.16 M precursor solution was prepared by dissolving stoichiometric PEAI and SnI_2 in a solvent mixture of DMF:DMSO (4:1) and stirred at room temperature. For the inks with additives, SnF_2 (0.016 M) and w $NaBH_4$(1 mM) $NaBH_4$, respectively, were added to the precursor solutions.

Inkjet printing and thermal treatments of PEA_2SnI_4 layer

The details of the printing procedure of the PEA_2SnI_4 are indicated as follows: the ink formulation was based on mixing DMF/DMSO solvents (4:1). An ink ejection frequency of 2.0 kHz and a resolution of 560 drops per inch (DPI) was used. During the PEA_2SnI_4 layer printing, the platen substrate temperature was kept constant at 20 °C in order to promote a proper spreading and adhesion among the different sublayers piled up to complete the thick full layer. After the inkjet-printing process, the layers were submitted to the curing process based on vacuum annealing at 110 °C for 15 minutes.

Device Fabrication

The PEDOT:PSS solution was filtered with 0.45 µm PVDF filter and spin coated on top of ITO at 5,000 rpm (2,000 rpm/s of acceleration) for 40 s and annealed at 130 °C for 20 min in ambient conditions. After the hole transporting layer (HTL) deposition the substrates were introduced in a N_2-filled glovebox, for the PEA_2SnI_4 inkjet printing layer deposition. The filtered solution of perovskite ink, with a viscosity around ~3 cP, was printed with a fixed drop-volume of 10 pL through 21-µm diameter nozzle by using cartridge for Dimatix printers (Fujifilm Dimatix Inc.) on top of PEDOT:PSS-coated layers. The preparation of the inks and the printing processes were both carried out in a glove box.

Finally, the substrates were transferred to a vacuum chamber, to evaporate 40 nm of PO-T2T, 1 nm of LiF and 100 nm of Al. The area of the flexible device was defined by the evaporation mask, which was 0.08 cm^2.

Inkjet-printed film/device characterization

The morphology and structure of Inkjet-printed perovskite thin films were studied by field emission scanning electron microscope (FEGSEM - JEOL 3100F) operated at 15 kV.

The crystalline microstructure, orientation and size of the NCs were determined by XRD using a Japan Rigaku D/Max-IIA X-ray diffractometer using Cu Kα radiation, l = 1.5406 Å, operating at 40 keV and 40 mA.

The optical properties were evaluated by measuring the transmittance and reflectance spectra, by using an integrating sphere (Bentham PV300 EQE system), using monochromated light from a Xe and quartz halogen dual lamp source through the 300–1100 nm range, and collecting the transmitted (or reflected) light with an InGaAs photodetector. Finally, the emission properties were determined by acquiring photoluminescence (PL) spectra of the films grown onto Si substrates, exciting the samples with the 325-nm line of a He-Cd laser, with a power density of 8×10^3 W cm^{-2}, and analyzing the emitted light with a single-grating monochromator coupled to a GaAs photomultiplier.

External quantal efficiency (EQE) measurements were performed with a QEPVSI-b Oriel system. Stability measurements were performed fxing the voltage

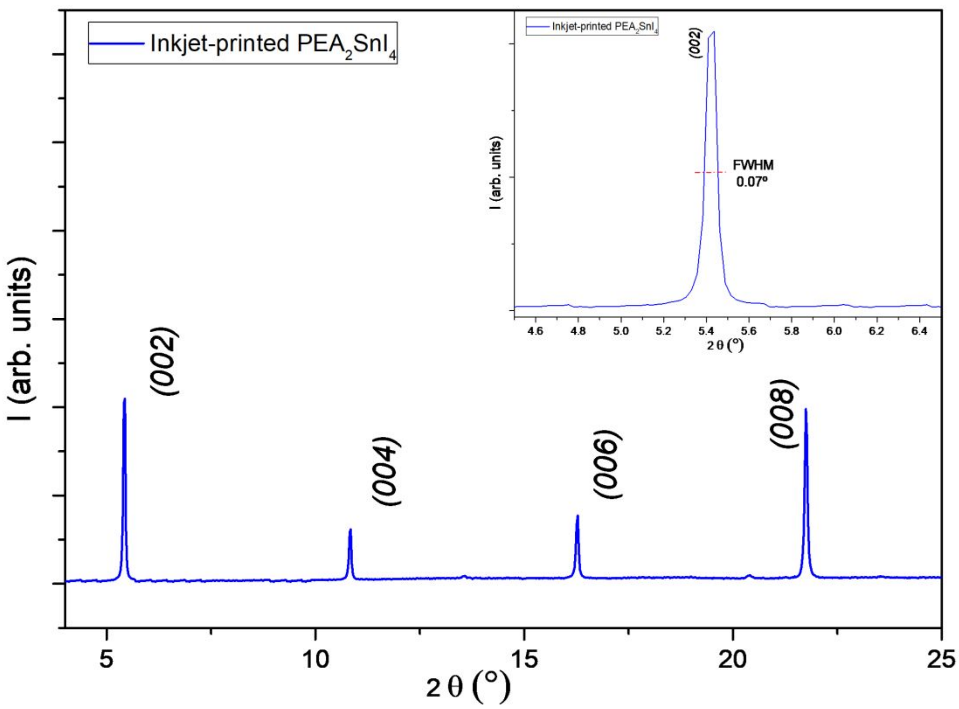

Figure S1 : XRD pattern of inkjet-printed PEA_2SnI_4 after vacuum annealing; detail of high-resolution XRD spectra (in the range from 4.5° to 6.5°) of PEA_2SnI_4 films (inset).

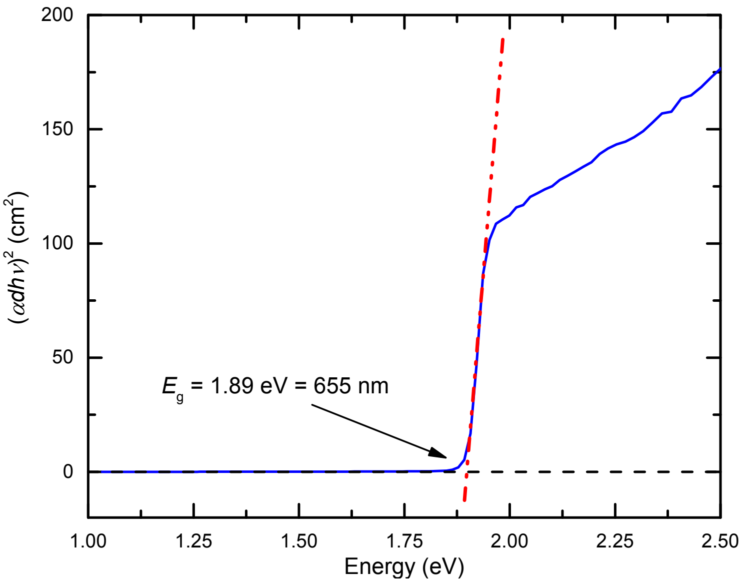

Figure S2: Tauc plot of inkjet-printed PEA_2SnI_4 after subtracting the excitonic emission.

In order to achieve the bet device structure, we selected PO-T2T as the electron-transporting acceptor due to its flat band energy level (−3.5 eV)[1,2] which matches well with the conduction band of PEA_2SnI_4 (−3.3 eV)[3]. Beside reduced losses at the ETL/perovskite interface, PO-T2T has a higher electron mobility (≈10^{-3} cm^2 V^{-1} s^{-1}), low refractive index (~1.72).[2,4] In the same way the PEDOT:PSS 4083 was selected for is high hole conductivity (10^{-2} to 10^{-3} S cm^{-1}).[5]

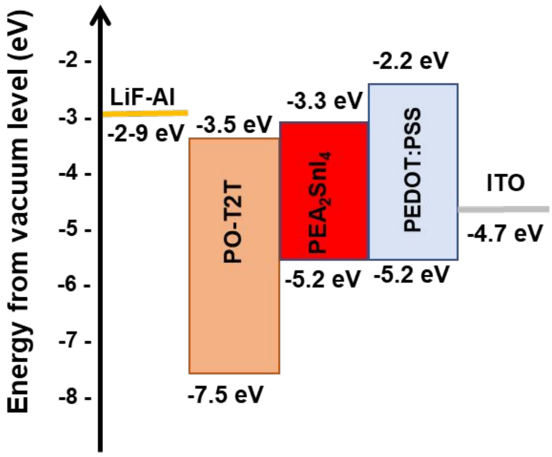

Figure S3: Energy level diagram.

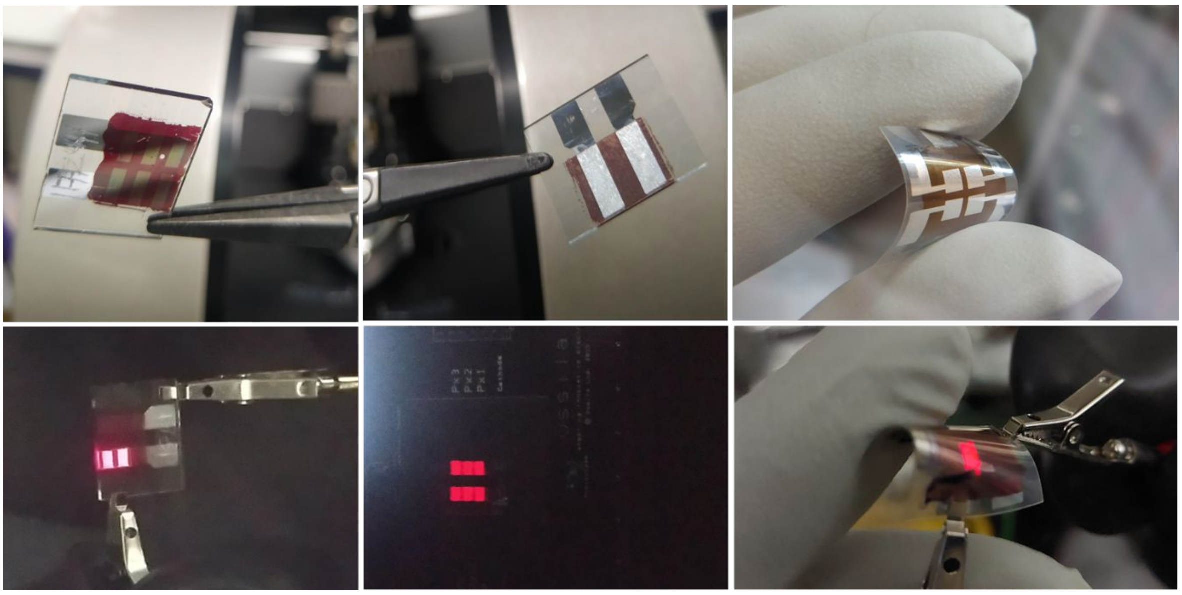

Figure S4: Inkjet printed PEA_2SnI_4 LEDs on 6 pixels (0.045 cm^2) glass substrate and on 4 pixels (0.08 cm^2) flexible PI.

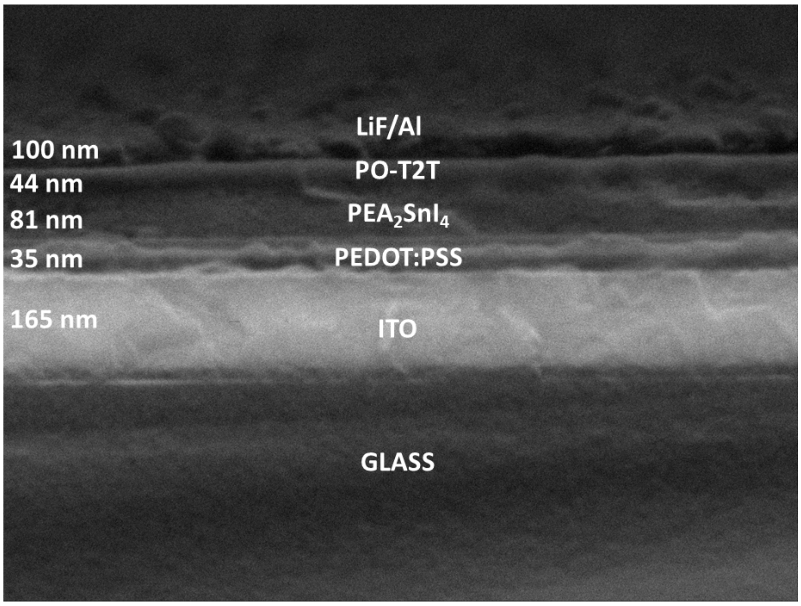

Figure S5: SEM cross section PeLED device.

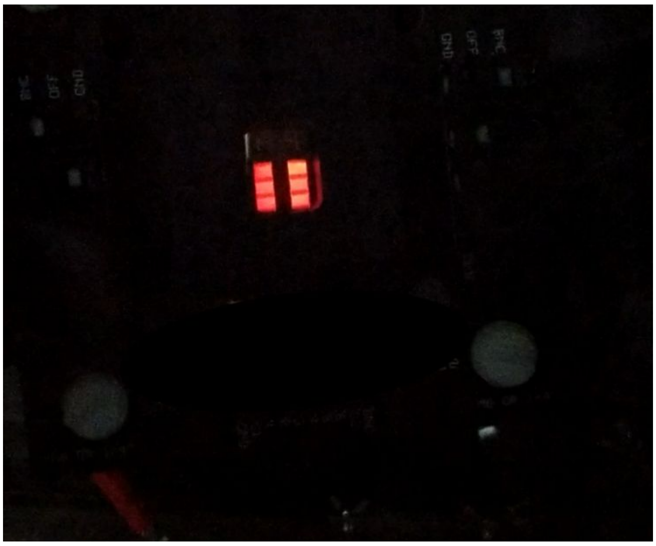

Figure SV1: Video of an array of 6 pixels LED red emitting based on 2D perovskite PEA_2SnI_4 under 4 V voltage applied.

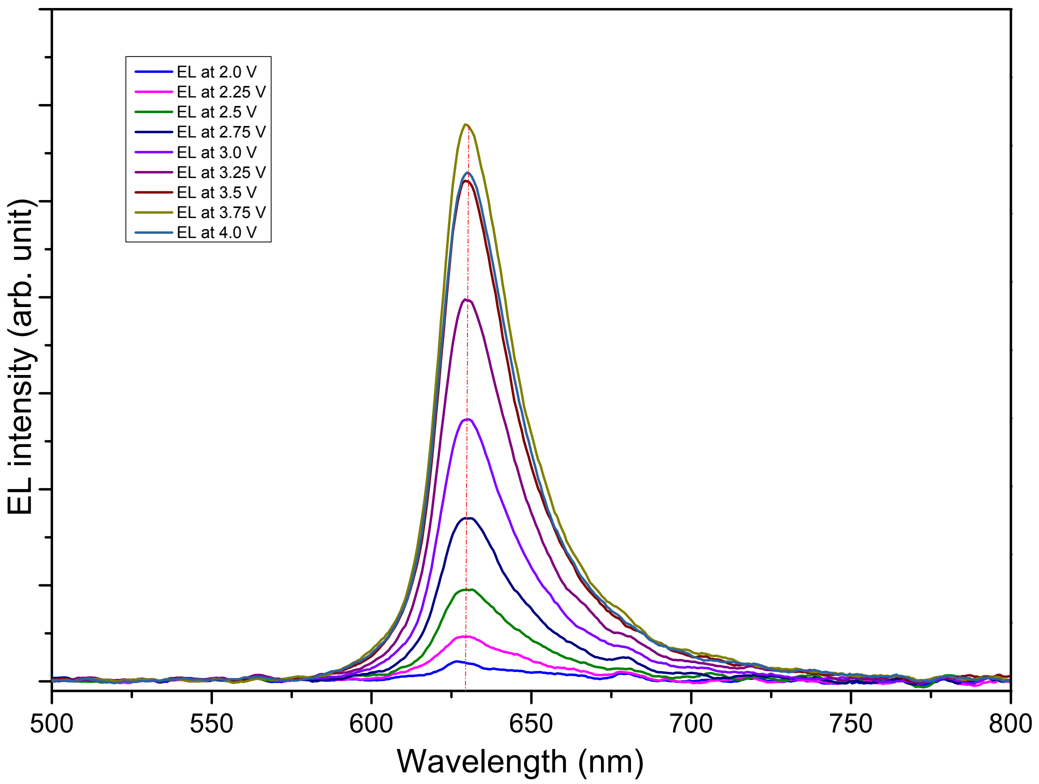

Figure S6: EL spectral characteristics of inkjet-printed PEA_2SnI_4 devices is red emitting with a peak centered at 630 nm with FWHM of 25 nm at different operation voltage.

Biblioraphy:

(1) Chin, X. Y.; Perumal, A.; Bruno, A.; Yantara, N.; Veldhuis, S. A.; Martínez-Sarti, L.; Chandran, B.; Chirvony, V.; Lo, A. S.-Z.; So, J.; Soci, C.; Grätzel, M.; Bolink, H. J.; Mathews, N.; Mhaisalkar, S. G. Self-Assembled Hierarchical Nanostructured Perovskites Enable Highly Efficient LEDs via an Energy Cascade. *Energy Environ Sci* **2018**, *11* (7), 1770–1778. https://doi.org/10.1039/C8EE00293B.

(2) Salim, K. M. M.; Hassanabadi, E.; Masi, S.; Gualdrón-Reyes, A. F.; Franckevicius, M.; Devižis, A.; Gulbinas, V.; Fakharuddin, A.; Mora-Seró, I. Optimizing Performance and Operational Stability of CsPbI3Quantum-Dot-Based Light-Emitting Diodes by Interface Engineering. *ACS Appl Electron Mater* **2020**, *2* (8), 2525–2534. https://doi.org/10.1021/acsaelm.0c00431.

(3) Yuan, F.; Zheng, X.; Johnston, A.; Wang, Y. K.; Zhou, C.; Dong, Y.; Chen, B.; Chen, H.; Fan, J. Z.; Sharma, G.; Li, P.; Gao, Y.; Voznyy, O.; Kung, H. T.; Lu, Z. H.; Bakr, O. M.; Sargent, E. H. Color-Pure Red Light-Emitting Diodes Based on Two-Dimensional Lead-Free Perovskites. *Sci Adv* **2020**, *6* (42), 253–267. https://doi.org/10.1126/sciadv.abb0253.

(4) Chin, X. Y.; Perumal, A.; Bruno, A.; Yantara, N.; Veldhuis, S. A.; Martínez-Sarti, L.; Chandran, B.; Chirvony, V.; Lo, A. S.-Z.; So, J.; Soci, C.; Grätzel, M.; Bolink, H. J.; Mathews, N.; Mhaisalkar, S. G. Self-Assembled Hierarchical Nanostructured Perovskites

Enable Highly Efficient LEDs via an Energy Cascade. *Energy Environ Sci* **2018**, *11* (7), 1770–1778. https://doi.org/10.1039/C8EE00293B.

(5) Wang, J.; Song, C.; He, Z.; Mai, C.; Xie, G.; Mu, L.; Cun, Y.; Li, J.; Wang, J.; Peng, J.; Cao, Y. All-Solution-Processed Pure Formamidinium-Based Perovskite Light-Emitting Diodes. *Advanced Materials* **2018**, *30* (39), 1804137. https://doi.org/10.1002/adma.201804137.
